# A silicon-on-insulator slab for topological valley transport

**DOI:** 10.1038/s41467-019-08881-z

**Published:** 2019-02-20

**Authors:** Xin-Tao He, En-Tao Liang, Jia-Jun Yuan, Hao-Yang Qiu, Xiao-Dong Chen, Fu-Li Zhao, Jian-Wen Dong

**Affiliations:** State Key Laboratory of Optoelectronic Materials and Technologies & School of Physics, Sun Yat-sen University, Guangzhou, 510275 China

## Abstract

Backscattering suppression in silicon-on-insulator (SOI) is one of the central issues to reduce energy loss and signal distortion, enabling for capability improvement of modern information processing systems. Valley physics provides an intriguing way for robust information transfer and unidirectional coupling in topological nanophotonics. Here we realize topological transport in a SOI valley photonic crystal slab. Localized Berry curvature near zone corners guarantees the existence of valley-dependent edge states below light cone, maintaining in-plane robustness and light confinement simultaneously. Topologically robust transport at telecommunication is observed along two sharp-bend interfaces in subwavelength scale, showing flat-top high transmission of ~10% bandwidth. Topological photonic routing is achieved in a bearded-stack interface, due to unidirectional excitation of valley-chirality-locked edge state from the phase vortex of a nanoscale microdisk. These findings show the prototype of robustly integrated devices, and open a new door towards the observation of non-trivial states even in non-Hermitian systems.

## Abstract (web summary)

Backscattering is one of the major factors that limit the performance of integrated nanophotonics. Here, He et al. realize topologically protected, robust and unidirectional coupling as well as optical transport on a silicon-on-insulator platform by exploiting the valley degree of freedom.

## Introduction

Silicon-on-insulator (SOI) provides a CMOS-compatible platform to fasten and enlarge data transfer both between and within silicon chips, by using optical interconnects to replace their electronic components^1^. Miniaturization of SOI devices can achieve highly integrated photonic structures comprised of numerous optical components in a single chip, but increase inevitable backscattering that leads to energy loss and signal distortion. Consequently, optical backscattering suppression is of fundamental interest and great importance for compact SOI integration. The discovery of topological photonics offers an intriguing way for robust information transport of light^2,3^, particularly for their capacities in backscattering–immune propagation and unidirectional coupling. Such robustness is derived from the nontrivial bulk topology, enabling reflection-free transport between two topologically distinct domains^2–17^, such as by using a magneto-optical effect, 3D chiral structures, and bianisotropic metamaterials. As a target to integrated topological nanophotonics, some all-dielectric strategies have been proposed recently. An array of coupling resonator optical waveguides was designed to implement topological SOI structures at super-wavelength period^18^, and has been exploited to the topological-protected lasing effect^19,20^. Later, a subwavelength-scale strategy attracted much attention to reduce the size of topological devices, e.g., applying *C*_*6v*_ crystalline symmetries to realize all-dielectric topological structures above a light cone^21–23^, which have been achieved under in-plane unidirectional propagation over sufficient distances^24^ and observed topological states through out-of-plane scattering^25^. These developments of topological nanophotonics open avenues to develop on-chip optical devices with built-in protection, such as robust delay lines, on-chip isolation, slow-light optical buffers, and topological lasers.

Valley pseudospin provides an additional degree of freedom (DOF) to encode and process binary information in graphene and two-dimensional transition metal dichalcogenide (TMDC) monolayers^26–29^. Analogous to valleytronics, exploration of valley physics in classical waves (e.g., photonics^30–37^ and phononics^38–41^) renders powerful routes to address the topological nontrivial phase by emerging an alternative valley DOF. To retrieve the topological valley phase, a general method is to break spatial-inversion symmetry for accessing the opposite Berry curvature profiles near Brillouin zone corners, i.e., the K and K′ valley. Advanced in nanofabrication techniques, precise manufacture of the inversion-symmetry-broken nanophotonic structures is easy to implement nowadays^42^. Consequently, valley photonic crystal (VPC) is a reliable candidate for SOI topological photonic structures, in particular for a subwavelength strategy that still remains much of a challenge in topological nanophotonics. Furthermore, the topological valley phase below a light cone ensures high–efficient light confinement in the plane of a chip, such that the photonic valley DOF naturally makes a balance between in-plane robustness and out-of-plane radiation. This is a crucial condition to design topological photonic structures for chips. Realization of topological valley transport in the SOI platform is desirable for integrated topological nanophotonics.

In this work, we experimentally demonstrate a valley topological nanophotonic structure at telecommunication wavelength. Our design is based on a standard SOI platform that allows integration with other optoelectronic devices on a single chip. Valley-dependent topological edge states can operate below light cone, benefiting to the balance between in-plane robust transport and out-of-plane radiation. Broadband robust transport is observed in sharp-turning interfaces constructed by two topologically distinct valley photonic crystals, with a footprint of 9 × 9.2 μm^2^. In addition, we achieve topological photonic routing with high directionality, by exploiting unidirectional excitation of valley–chirality-locked edge state with a subwavelength microdisk.

## Results

### Silicon-on-insulator valley photonic crystals

**Fig. 1:**
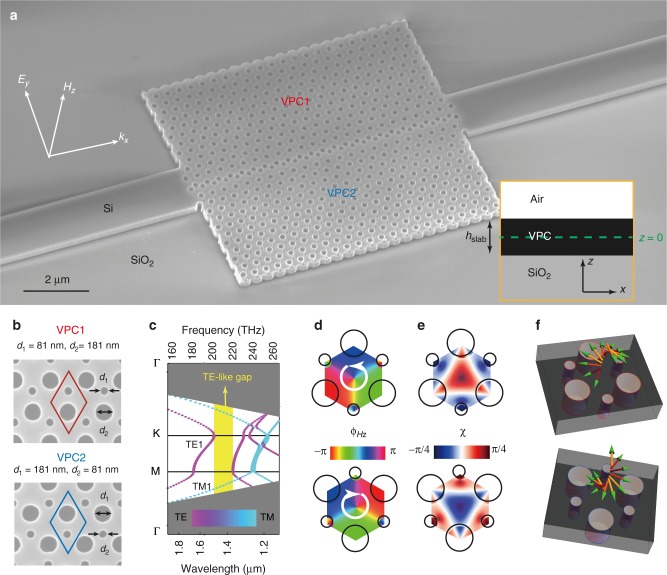
Band structures and nontrivial topology in silicon-on-insulator (SOI) valley photonic crystals (VPCs). **a** Oblique-view scanning-electron-microscope image of the fabricated VPC, which is patterned on standard 220-nm-thickness silicon wafer. The VPC is arranged in a honeycomb lattice with a periodicity of *a* = 385 nm. The inset indicates that the VPC slab is asymmetrically placed between the SiO_2_ substrate and the top air region along the *z* axis. **b** Details of the unit cells consisting of two inequivalent air holes, i.e., the smaller one with a diameter *d*_1_ (*d*_2_) = 81 nm and the larger one with a diameter *d*_2_ (*d*_1_) = 181 nm for VPC1 (VPC2). **c** Bulk band both for VPC1 and VPC2. The colormap indicates the linear polarization of a photonic band. There is a 132-nm-bandwidth gap (yellow region) between the first and second TE-like bands (purple) due to the *y*-axis inversion symmetry broken. Gray region: light cone of silica. **d** Simulated phase vortex of *H*_*z*_ field profile and **e** ellipticity angle of (*E*_*x*_, *E*_*y*_) field at the K valley of the TE1 band for VPC1 and VPC2. The *H*_*z*_ phase vortex mainly rotates along the unit cell center, of which the singularity point corresponds to right- or left-handed circular polarization (RCP or LCP). All of the field patterns shown below focus on the *z*-central plane (labeled as *z* = 0 in the inset of Fig. 1a). **f** Temporal evolution of RCP and LCP at the singularity point, respectively. In this work, the refractive index of silicon and silica are *n*_Si_ = 3.47 and *n*_Silica_ = 1.45, respectively

In this work, our nanophotonic structures are prepared on SOI wafers with 220-nm-thickness silicon layers. As depicted in Fig. 1a, the valley photonic structure comprises two honeycomb photonic crystals (VPC1 and VPC2). The VPC layer is asymmetrically placed between the SiO_2_ substrate and top air region along the *z* axis (see the inset of Fig. 1a). Instead of a freestanding membrane, the use of a *z*-asymmetric SOI slab can improve the compatibility with other types of building blocks (e.g., microdisk later in this work). Figure 1b gives the details of VPC. The unit cell of VPC1 (red) contains two nonequivalent air holes, i.e., the smaller one *d*_1_ = 81 nm and the bigger one *d*_2_ = 181 nm. On the other hand, the diameter of two air holes is altered to form another type of VPC (blue), i.e., VPC2 with *d*_1_ = 181 nm and *d*_2_ = 81 nm. Here, VPC2 is the inversion-symmetry partner of VPC1. Thus, VPC1 and VPC2 have the same band structure, as shown in Fig. 1c. The details about the VPC design can be seen in Supplementary Note 1. Because the two air holes have different diameters that break the inversion symmetry, a bandgap (1360 nm ~1492 nm) emerges for TE-like polarizations.

Due to the bulk-edge correspondence, we will first study the bulk states of the first TE-like band (labeled as “TE1” in Fig. 1c), before discussing the topology of a TE-like gap. The electromagnetic fields in the *z*-central plane (labeled as “*z* = 0” in the inset of Fig. 1a) can mainly reflect the optical properties of the VPC slab, so that we will focus on the field patterns at *z* = 0 plane in the following discussion. Take the eigenstates at the K valley as examples. The simulated *H*_*z*_ phase profile at *z* = 0 plane is plotted in Fig. 1d. We can see that the phase profile of VPC1/VPC2 increases anticlockwise/clockwise by 2π phase around the center of a unit cell. Such optical vortex is related to valley pseudospin in an electronic system, and thus can be termed as a photonic valley DOF. In TMDCs, the valley-polarized excitons can be selectively generated through control of the chirality of light^43,44^. Similarly, the photonic valley is also locked to the chirality of excited light, i.e., left-circularly polarized (LCP) light couples to the K′-valley mode, while right-circularly polarized (RCP) light couples to the K-valley mode^32^. To demonstrate this valley–chirality locking property remaining in the SOI slab, we give the distribution of the polarization ellipse of the in-plane electric field in Fig. 1e. Here, the polarization ellipse is generally defined as ellipticity angles^45^ $\chi = \arcsin [ {2| {E_x} || {E_y} |\sin \delta / ( {| {E_x} |^2 + | {E_y} |^2} )} ]/2$, where $\delta = \delta _y - \delta _x$ is the phase difference between *E*_*y*_ and *E*_*x*_. For VPC1, the RCP response (*χ* = π/4) exists in the singularity point of the phase vortex at the K valley (red center in Fig. 1e), and vice versa for VPC2 (blue center in Fig. 1e). Figure 1f shows the temporal evolution of the RCP and LCP responses, respectively. Such valley–chirality locking gives the possibility to manipulate photonic valley modes. For example, when we place a circular-polarized dipole source in the singular point of the phase vortex, the photonic valley Hall effect can be observed (see Supplementary Note 2).

**Fig. 2:**
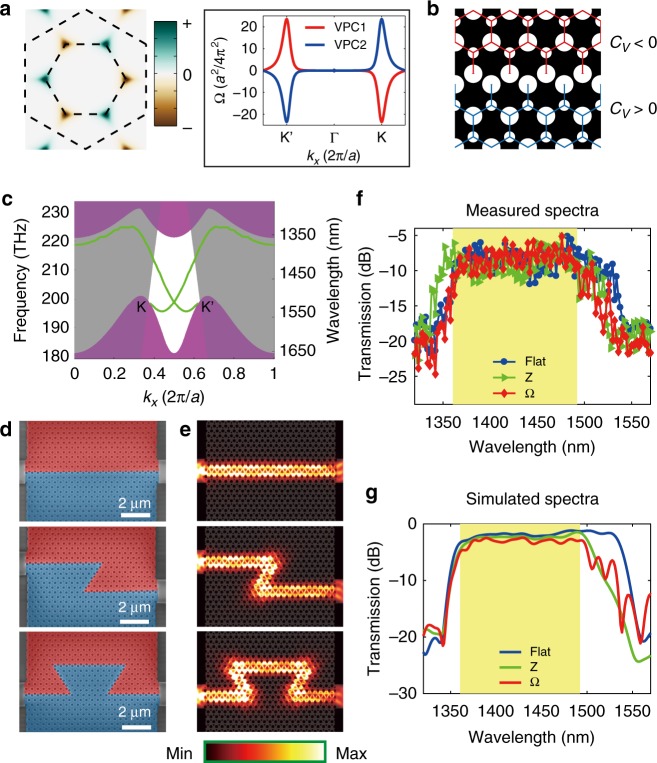
Valley-dependent topological edge states in silicon-on-insulator (SOI). **a** Distribution of TE1 Berry curvature for VPC1, which is localized near the corners of the first Brillouin zone. The valley-dependent Berry curvature has opposite sign to each valley. The inset plots the Berry curvature along *k*_*y*_ = 0 direction, indicating that the Berry curvature of VPC2 (blue) has inversed distribution to that of VPC1 (red). **b** Schematic of the valley-dependent topological interface constructed by two types of valley photonic crystal (VPC) slabs. The VPC1 (red) and VPC2 (blue) attribute to different topological phases, characterized by the sign of their valley Chern index *C*_*V*_. **c** Dispersion of the valley-dependent edge states (green lines) for the bearded-stack interface. Purple region: projection TE-like band. Translucent gray region: light cone of silicon dioxide substrate. **d** Top-view scanning-electron-microscope images of the fabricated samples, including flat, Z-shape, and Ω-shape topological interfaces. **e** Simulation of electromagnetic energy intensities for the three distinct shape interfaces at *z* = 0 plane (*λ*  =1430 nm). The Z- (Ω-) shape interface with two (four) 120^o^ bends shows that light can smoothly propagate around the corners. **f** Measured and **g** simulated transmission spectra for the flat (blue), Z-shape (green), and Ω-shape (red) interfaces, respectively. The yellow regions correspond to the TE-like gap of SOI VPC. All of the spectra in the bandgap maintain the flat-top high transmittance, even for a sharp-bending geometry (green and red). This intriguing property indicates broadband robust transport in the frequency interval from 1360 to 1492 nm

The above observation of the optical vortex and the photonic valley Hall effect can be related to a topologically nontrivial phase. Next, we numerically calculate the Berry curvature distribution and the corresponding topological invariant to insightfully confirm the topological valley phase in our proposed VPC slab^46^. See the section Methods for more details on numerical simulation. Figure 2a shows the Berry curvature of the TE1 band for VPC1, calculated with the parallel gauge transformation^47^. The Berry curvature of VPC is mainly distributed near two valleys, i.e., a singular sink at the K valley while peaking at K′ (red line in the inset of Fig. 2a). On the contrary, VPC2 reverses the Berry curvature distribution of the two valleys (blue line in the inset of Fig. 2a). In general, the global integration of Berry curvature over the whole Brillouin zone, the so-called Chern number, is zero under the protection of time-reversal symmetry. Instead, the valley-dependent integration of Berry curvature gives rise to a nonzero value, i.e., the valley-dependent index *C*_K_ (*C*_K′_) ≠ 0. Thus, we can use the valley Chern index *C*_*V*_ = *C*_K_–*C*_K′_ to characterize the topology of the whole VPC system, providing a new route to retrieve a topologically nontrivial phase.

We should note that the VPCs open a large TE-like gap (~10%) to guarantee broad bandwidth operation. Thus, the Berry curvature for both valleys will overlap with each other. As a consequence, the valley Chern index is not a well-defined integer, i.e., $0 < \left| {C_V} \right| < 1$. The edge dispersion will not gaplessly cross from the lower band to the upper band. Regardless of this side effect, the difference in the sign of valley Chern index will ensure the protection of the topological valley phase, as long as the bulk state at the K valley is orthogonal to the K′ valley. Therefore, such novel design still enables broadband robust transport along the ΓK/ΓK′ direction against certain perturbations (such as sharp-bend corners or 10% random bias of hole diameter), as the intervalley scattering is suppressed due to the vanishing field overlapping between two valley states.

As an intuitive example, we construct an interface by using two VPCs with the opposite valley Chern index. As schematically shown in Fig. 2b, the bearded interface is stacked with the bigger holes. The upper domain (VPC1 in Fig. 1) has valley Chern index *C*_*V*_ < 0, while the valley Chern index of the lower domain (VPC2 in Fig. 1) exhibits the opposite sign (*C*_*V*_ > 0). The valley-dependent edge states (green lines in Fig. 2c) for the bearded-stack interface, include one with negative velocity at the K valley and the other with positive velocity at the K′ valley. The simulated patterns shown in Fig. 2e confirm that the propagating light at *λ* = 1430 nm will smoothly detour by 120^o^ bending (60^o^ sharp corner). Such propagation is valid for other wavelengths inside the bandgap, leading to optical broadband operation. Note that a little bit modulation of the signal in Fig. 2e is mainly caused by the out-of-plane radiation loss in the open-slab system. Note also that the TE/TM coupling in such *z*-asymmetric slab is too weak (see Supplementary Note 3) to affect valley-dependent interface transport, i.e., the robust transport in 120^o^ bending and unidirectional coupling. In the next section, we will experimentally characterize this broadband robust transport phenomenon.

### Topological robust transport

To experimentally demonstrate topological robust transport of the valley-dependent edge states, we employ an advanced nanofabrication technique to manufacture the flat-, Z-, and Ω-shape VPC interfaces. The scanning-electron-microscope (SEM) images of fabricated samples are shown in Fig. 2d. The devices were prepared on a SOI wafer, with a nominal 220-nm silicon layer and 2.0-μm buried oxide layer. After the definition of a 370-nm-thickness positive resist through electron-beam lithography, inductively coupled plasma etching step is applied to pattern the top silicon layer, such that the VPC structure and its coupling waveguide was formed. Then the resist was removed by using an ultrasonic treatment process. See Methods for more details of the nanofabrication process. These processes are able to precisely achieve our designed structures even in close proximity (separation of about 40 nm in the topological interface).

Next, we will characterize the broadband robust transport of the VPC edge states at the Z/Ω-shape interface. The experimental setup is shown in Supplementary Figure 5. The TE-polarized continuous waves at the telecommunication wavelength were coupled to the 1.7-μm-width input waveguide by using a polarization-maintaining lensed fiber, and then launched into the VPC sample from the left end of the topological interface. After passing through the VPC devices, the propagating wave was coupled to the output waveguide at the right end and then collected by another lensed fiber. The corresponding transmission spectra were detected by using an optical powermeter, with tuning the operation wavelength of excited waves. Note that all the transmission spectra are normalized to the 1.7-μm-width silicon strip waveguide located in the same writing field near the VPC samples. See Methods for more details of optical characterizations. Figure 2f shows the measured transmission spectra in the wavelength range of 1320–1570 nm, for flat-, Z-, and Ω-shape topological interfaces. In the bandgap region (yellow), the spectra are kept on the flat-top high-transmittance platform, even for a sharp-bending geometry (green and red lines). This intriguing property indicates the broadband robust transport in the frequency interval from 1360 to 1492 nm, due to the suppression of intervalley scattering. Although there is some noise in Fig. 2f due to Fabry–Perot resonance between the entrance and exit facets of the strip waveguide and dark current noise of the detector, these experimental spectra are in good agreement with simulations (Fig. 2g). Note that the SOI platform with a compact footprint of 9 × 9.2 μm^2^ enables to integrate many photonic components on a single chip. In other words, the proposed SOI VPC with a subwavelength periodicity (about *λ*/4) can develop a high-performance topological photonic device with a compact feature size of less than 10 μm.

### Unidirectional coupling

**Fig. 3:**
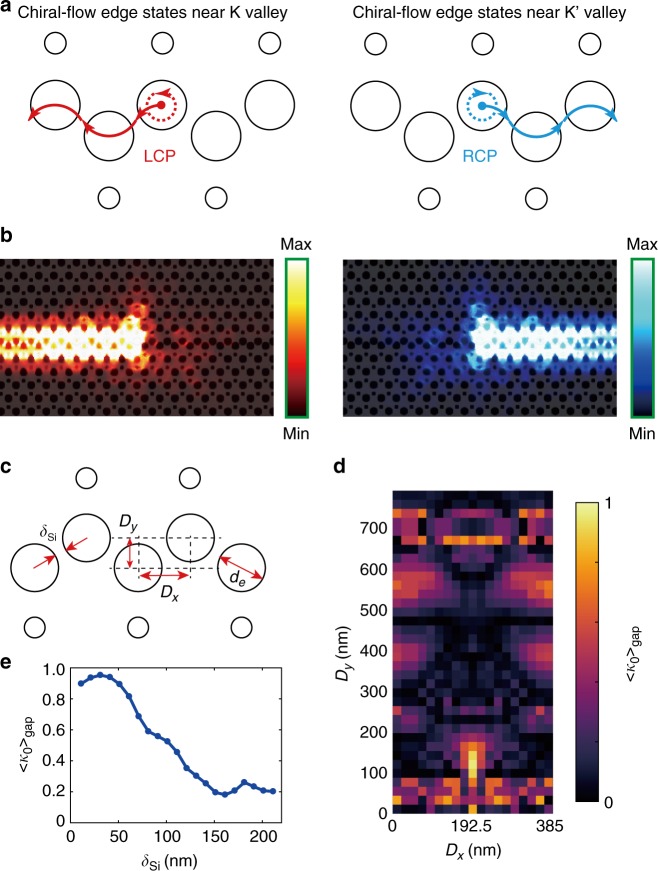
Unidirectional coupling of valley-dependent edge states by using a circularly polarized chiral source. **a** Illustration of unidirectional coupling along the valley photonic crystal (VPC) interface, based on the selective excitation of the phase vortex. The vortex fields around the bearded holes at the upper domain will interact with those of the lower domain, and thus a chiral-flow edge state occurs. **b** Simulation of unidirectional coupling in a bearded-stack interface by controlling the chirality of a circularly polarized source at *λ* = 1430 nm. To simplify, a right-circularly polarized (RCP) or left-circularly polarized (LCP) dipole source is considered to generate a clockwise/anticlockwise phase vortex. **c**–**e** Analysis of unidirectional coupling efficiency determined by an averaged parameter $\left\langle {\kappa _0} \right\rangle _{gap}$. Here, $\left\langle {\kappa _0} \right\rangle _{gap}$ is related to the frequency-domain integration of directionality $\kappa _0$ in the whole bandgap. **c** Key geometry parameters for global directionality, including relative location (*D*_*x*_ and *D*_*y*_) and diameter (*d*_*e*_) of bearded edge holes. The separation (*δ*_Si_) of the silicon region between the two bearded holes is variable as $\delta _{{\mathrm{Si}}} = \sqrt {D_x^2 + D_y^2} - d_e$. **d** Simulated phase map of $\left\langle {\kappa _0} \right\rangle _{gap}$ varied with relative locations *D*_*x*_ and *D*_*y*_. The maximum value emerges at the point of *D*_*x*_ = 192.5 nm and *D*_*y*_ = 111 nm, which is in correspondence with the valley-dependent topological interface. **e** Global directionality $\left\langle {\kappa _0} \right\rangle _{gap}$ as a function of separation *δ*_Si_ when tuning the diameters *d*_*e*_. Here, the relative position is fixed with the maximum point of Fig. 3d

Unidirectional coupling is another important property of topological photonic structures to manipulate the flow of light. We should emphasize that the realization of robust transport does not definitely correspond to unidirectional coupling. For example, it is inaccessible to achieve high-efficiency unidirectional coupling from a single circularly polarized source to zigzag-stack valley-dependent interfaces, due to protection of the inversion symmetry with regard to the *y*-axis center of the interface. In such *y*-odd/even-like edge states, the circular-polarized point of the polarization ellipse is predominant at the low-intensity positions. For high-efficiency unidirectional coupling, breaking the inversion symmetry of the interface is required to engineer the generation of vortex fields in the edge states. Therefore, we chose bearded-stack VPC interface (Fig. 2b) with the inversion symmetry broken. In this case, the vortex fields around bearded holes at the upper domain will interact with the lower domain, and thus generate chiral-flow edge states (see Supplementary Note 3). As depicted in Fig. 3a, such chirality ensures the rightward (leftward) excitation by using the RCP (LCP) source. Simulated results in Fig. 3b confirm that the proposed valley-dependent topological interface can realize unidirectionality through control of the source chirality. In fact, similar results have been studied in a photonic crystal W1-like waveguide, by shifting one side of the waveguide by half a lattice constant^48,49^.

We should emphasize that the introduction of a topological nontrivial phase can guarantee high directionality under chiral source excitation in relatively broadband operation, while the case of the topologically trivial system commonly operates in a narrowband as it is sensitive to the source position with frequency variation. To quantitatively determine unidirectional coupling, we define the directionality for a given frequency as $\kappa _0 = \left( {T_L - T_R} \right)/\left( {T_L + T_R} \right)$, where *T*_*L*_ and *T*_*R*_ are the transmittances detected at the left and right end, respectively. Furthermore, we would like to analyze the global efficiency of unidirectional coupling inside the photonic bandgap, and thus define an averaged parameter related to the frequency-domain integration of $\kappa _0$ in the whole bandgap, i.e., $\left\langle {\kappa _0} \right\rangle _{{\mathrm{gap}}} = | {{\int}_{{\mathrm{gap}}} {\kappa _0d\omega } } |/\Delta \omega _{{\mathrm{gap}}}$, where $\Delta \omega _{{\mathrm{gap}}}$ is the bandwidth of a photonic bandgap. $\left\langle {\kappa _0} \right\rangle _{{\mathrm{gap}}} = 1$ represents that the chiral source couples to pure left-/right-forward edge states for all frequencies in the bandgap. Here, we analyze the global directionality in the center on two dominant factors, i.e., relative positions (*D*_*x*_ and *D*_*y*_) and diameter (*d*_*e*_) of bearded edge holes (Fig. 3c). The separation (*δ*_*Si*_) of the silicon region between the two bearded holes is variable as $\delta _{{\mathrm{Si}}} = \sqrt {D_x^2 + D_y^2} - d_e$. A simulated phase map of Fig. 3d shows that the maximum $\left\langle {\kappa _0} \right\rangle _{{\mathrm{gap}}}$ emerges at the point of *D*_*x*_ = 192.5 nm and *D*_*y*_ = 111 nm, which is in correspondence with the valley-dependent topological interface. It is interesting that the proposed design based on valley topology can certainly find the point of high directionality, while the general method requires massive simulations, just like what we do in Fig. 3d.

On the other hand, considering a fixed relative position that *D*_*x*_ = 192.5 nm and *D*_*y*_ = 111 nm, the global directionality $\left\langle {\kappa _0} \right\rangle _{{\mathrm{gap}}}$ as a function of separation *δ*_*Si*_ is also retrieved in Fig. 3e, when tuning the diameters *d*_*e*_ of bearded edge holes. We can see that $\left\langle {\kappa _0} \right\rangle _{{\mathrm{gap}}}$ will stand on a high-directionality platform (above 0.9), when the separation is less than 50 nm. Qualitatively, this is because such extreme separation will enhance the interaction of vortex fields between upper- and lower-domain bearded holes, and thus strengthen the valley–chirality coupling of the topological interfaces.

### Topological photonic routing

**Fig. 4:**
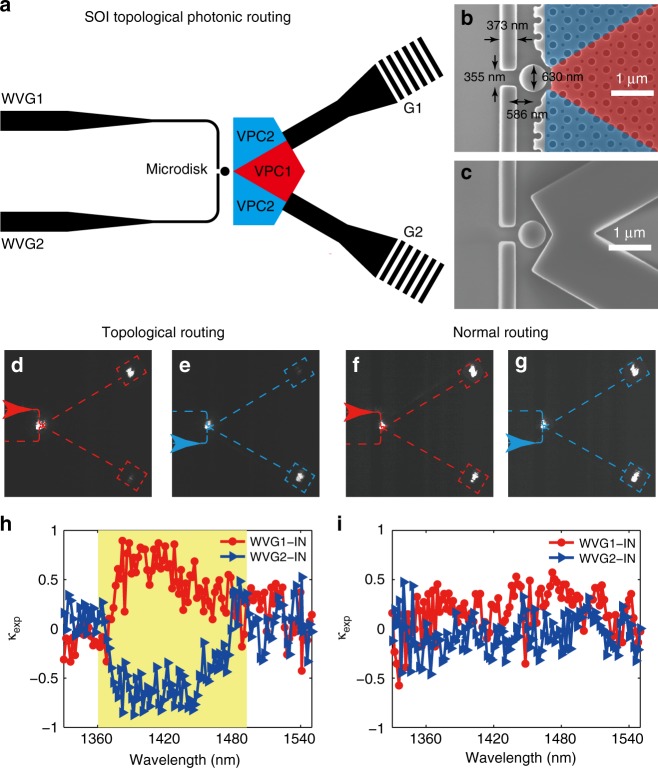
Experimental realization of topological photonic routing. **a** Schematic view of a topological photonic routing sample, including the valley photonic crystal (VPC) interface and the microdisk. The incident light from WVG1/WVG2 will excite an anticlockwise/clockwise phase vortex in the microdisk and then couple to the upper/lower interface in support of the different valley–chirality-locked modes. The light will finally couple back to free space by a nonuniform grating coupler (G1 and G2). **b** Scanning-electron-microscope image of the sample near a microdisk. The width of the input waveguide is *w*_*wvg*_ = 373 nm, and the air gap in the waveguide is *w*_*gap*_ = 355 nm. The diameter of microdisk *d*_*m*_ = 630 nm, with its distance to the center of the waveguide *h* = 586 nm. **c** Control sample with the same configuration to **b**, except for replacing the VPC interfaces by two-strip silicon waveguides. **d**–**g**, Measurement of photonic routing profiles at *λ* = 1400 nm, imaged by an optical far-field microscopy (20× objective). For topological routing (**d**, **e**), the incident light from the WVG1 port was routed to the upper interface, while another case from the WVG2 port was reversed. For normal routing (**f**, **g**), the light energy splits almost equally into two ports. **h**–**i**, Measured directionality spectra for topological (**h**) and normal (**i**) photonic routing devices. The spectra show the directional coupling efficiency, *κ*_*exp*_ = (*I*_*G1*_ –*I*_*G2*_)/(*I*_*G1*_ + *I*_*G2*_), as a function of the operation wavelength. Here, *I*_G1_ and *I*_G2_ are the extracted intensities collected from G1 and G2. For both WVG1 (red) and WVG2 (blue) incident case, the directional coupling efficiency of the topological routing device is beyond the value of 0.5 within a broadband region, some of which is close to unity. **i** For comparison, the normal routing device has low directionality within the considered wavelength range

Experimental realization of unidirectional coupling of topological edge states shows many promising applications in light manipulation. Recently, it has been demonstrated in the microwave region as a valley filter^36^ and realized in a chip-scale system as a topological quantum optics interface^24^. For the on-chip strategy, the latter one use chiral quantum dots under a strong magnetic field at ultralow temperature^24,50^. In this work, we aim to develop an all-optical strategy, for unidirectional excitation of the valley–chirality locking edge states in the SOI platform. To do this, a subwavelength microdisk serving as a phase vortex generator^51^, is introduced into the topological interface. Figure 4a shows the schematic of a designed device, combining SOI VPC and a microdisk. The fabricated sample around a microdisk can be seen in Fig. 4b. There are two 373-nm-width strip silicon waveguides (labeled as “WVG1” and “WVG2”) at the left of the sample. When incident light couples to the WVG1/WVG2 input waveguide, it will generate an anticlockwise/clockwise phase vortex at the designed microdisk with a close-to-diffraction-limited scale (630-nm diameter). Due to valley–chirality locking, the edge state near the K/K′ valley can be selectively routed to the upper/lower topological interface, through control of the chirality of the optical vortex inside a microdisk. This shows a prototype of the on-chip photonic routing device, with the advantage of an ultracompact (in sub-micrometer scale) coupling distance.

Far-field microscopy is used to verify the photonic valley–chirality locking property and the topological routing effect. A 20× objective is used to predominantly collect the out-of-plane radiation from two nonuniform grating couplers (labeled as “G1” and “G2” in Fig. 4a) and then imaged by using an InGaAs CCD. For a given incidence waveguide, an asymmetric radiation is obvious between G1 and G2. For example, the microscope images are presented in Fig. 4d for the WVG1 incidence at *λ* = 1400 nm. In this case, the propagating light was routed to the upper interface and radiated from the G1 port. The asymmetry of photonic routing is reversed when the incidence port is flipped to the other interface (Fig. 4e). For comparison, we also fabricated a control sample that replaced the SOI VPC by a two-strip silicon waveguide (Fig. 4c). The near-equal routing profiles demonstrate low directionality in Figs. 4f, g. Valley-dependent unidirectional routing is already visible to be distinguished from the control experiment.

The intensity of each grating coupler was collected from CCD, and the intensities *I*_*G1*_ and *I*_*G2*_ scattered from the upper (G1) and lower (G2) ports can be extracted with a high signal-to-noise ratio, after subtracting the noise that mainly arises from background radiation. The extracted intensities *I*_*G1*_ and *I*_*G2*_ reflect the amount of light transmission that is coupled to the upper- and lower-propagating valley-dependent edge states, respectively. To experimentally qualify directional coupling efficiency of routing devices, we define the experimental directionality as $\kappa _{\exp } = \left( {I_{G1} - I_{G2}} \right)/\left( {I_{G1} + I_{G2}} \right)$. The full-band directionality can be measured by tuning the operation wavelength of the excited waves. Figure 4h shows the directionality spectra as a function of wavelength. In the bandgap, a strong and broadband directionality was observed. For anticlockwise-phase-vortex excitation, the incident light couples to valley-dependent edge states propagating along the upper interface (red line in Fig. 4g). The directionality of the topological routing device is up to 0.5 within a broadband region. Note that the maximum $\kappa _{\exp }$ is up to ~0.895, implying a 18:1 extinction ratio between G1 and G2. When the handedness of the excitation flips, so do the propagation directions of the valley-dependent edge states (blue line in Fig. 4h). For comparison, the low-directionality spectra for the control experiment were depicted in Fig. 4i. There are a few discrete wavelengths to reach $| {\kappa _{\exp }} |$ > 0.5. A more experimental description is presented in Supplementary Note 5.

## Discussion

In summary, we have successfully applied the valley DOF to topologically manipulate the flow of light in a silicon-on-insulator platform. Benefiting from the below-light-cone operation, the valley-dependent topological edge state can balance in-plane robust transport and out-of-plane radiation, which is important to the open system, such as a photonic crystal slab. Topological robust transport and topological photonic routing are experimentally demonstrated and confirmed at telecommunication wavelength. Our study paves the way to explore the photonic topology and valley in the SOI platform, which is a promising system in taking advantage of the topological properties into nanophotonic devices, particularly important for backscattering suppression and unidirectional coupling. Furthermore, our subwavelength strategy enables to design compact-size topological SOI devices that allow integration with other optoelectronic devices on a single chip. It shows a prototype of the on-chip photonic device, with promising applications for delay line, routing, and dense wavelength division multiplexing for information processing based on topological nanophotonics. Finally, the platform of the SOI topology opens a new door toward the observation of nontrivial states even in non-Hermitian photonic systems.

We are aware of a related work on experimental demonstration of valley-dependent edge states through air-bridge slab structures with sharp-turning profiles^37^.

## Methods

### Numerical simulation

All of the simulation results in this work are retrieved from a 3D asymmetric slab instead of a 2D model^52^. The band structures and the corresponding eigenfield patterns were calculated by MIT Photonic Bands^53^ (MPB) based on the plane-wave expansion (PWE) method, while all of the optical transport calculations were implemented by MIT Electromagnetic Equation Propagation^54^ (MEEP) based on the finite-difference time-domain (FDTD) method. In all 3D simulations, the maximum scale of the discrete grid is smaller than 24 nm, making the resolution large enough to ensure the convergence. For Berry curvature calculations, the original data of eigenfield ${\bf{\psi }}\left( {x,y,z} \right) = \left[ {\sqrt {\varepsilon _z\left( {x,y,z} \right)} u_k^{Ez}\left( {x,y,z} \right),\sqrt {\mu _z\left( {x,y,z} \right)} u_k^{Hz}\left( {x,y,z} \right)} \right]^T$ are obtained from MPB, by scanning the whole Brillouin zone with step *δk* = 0.005(2*π/a*). Here, $u_k^{Ez}$ and $u_k^{Hz}$ are the periodic parts of *E*_*z*_ and *H*_*z*_, respectively. Then the Berry curvature can be calculated by ${\bf{\Omega }} = i{\bf{\nabla }}_{\bf{k}} \times \left\langle {\bf{\psi }} \right|{\bf{\nabla }}_{\bf{k}}\left| {\bf{\psi }} \right\rangle$.

### Sample fabrication

The experimental samples were manufactured by employing a top–down nanofabrication process on a SOI wafer (with a nominal 220-nm device layer and a 2.0-μm buried oxide layer). First, a 370-nm-thickness positive resist (ZEP520A) was spun with a rotating speed of 3500 min^−1^ on the wafer, and dried for 10 min at 180 °C. The VPC patterns were defined by electron-beam lithography (EBPG5000 ES, Vistec) in the resist, and developed by dimethylbenzene for 70 s. Second, inductively coupled plasma (ICP) etching step was applied to etch the VPC structures and coupling waveguides on the top 220-nm-thickness silicon layer. Then the resist was removed by using an ultrasonic treatment process at room temperature. The final step was to cut up and polish the facets of samples, in order for high–efficient incident coupling.

### Optical characterization

The experimental results of the transmission spectra and far-field microscopy images were realized with three tunable continuous-wave lasers (Santec TSL-550/710) at telecom wavelength (1260 ~1640 nm). The incident light was first launched into a fiber polarization controller to select the TE wave, and then coupled to the input waveguide with the aid of a polarization-maintaining lensed fiber. After passing through VPC devices, the propagating waves coupled to the output waveguide at the right end of the topological interface. For robust transport measurement (Fig. 2), the output signals were collected by another lensed fiber and detected by an optical powermeter (Ophir Nova-II). For photonic routing measurement (Fig. 4), the propagating waves coupled out in the *z*-direction thanks to the gratings at the end of the waveguide. The out-of-plane radiation was collected by a 20× microscope objective and then imaged by using an InGaAs CCD (Xenics Bobcat-640-GigE). More details on the experimental setup are provided in Supplementary Note 4.

## Supplementary information

Supplementary information

## Data Availability

The data that support the findings of this study are available from the corresponding author upon reasonable request.
