# Supplementary material for: A silicon-on-insulator slab for topological valley transport
Source: Nat Commun. 2019 Feb 20;10:872. doi: 10.1038/s41467-019-08881-z (PMC6382878; doi:10.1038/s41467-019-08881-z)
Supplement: Supplementary file 1 — Supplementary information [file 41467_2019_8881_MOESM1_ESM.pdf]

Supplementary Information

A silicon-on-insulator slab for topological valley transport

He et al.

Supplementary Note 1: Topological phase transition with inversion-symmetry broken

When the honeycomb lattice has two equivalent air holes ($d_1 = d_2 = 140$ nm), TE-like polarized Dirac point is present at the K/K' valley of the Brillouin zone due to the protection of C_{3v} point group symmetry, as shown in Supplementary Figure 1a. Such degenerate point will split into two non-degenerate eigenstates with *y*-axis inversion symmetry broken ($d_1 = 81$ nm and $d_2 = 181$ nm), such that the photonic band opens up a TE-like bandgap (Supplementary Figure 1b).

To demonstrate the phase transition, Supplementary Figure 1c shows the valley phase diagram, revealed by the band-edge frequencies at K valley as a function of δd. Here, $\delta_d = d_1 - d_2$ is the diameter difference between the upper and lower holes. The purple/green line of valley phase is locked with anticlockwise/clockwise vortex, which can be viewed as a pseudospin analogous to the A-B sublattice in graphene system [1]. The red (blue) dots represent the designed VPC1 (VPC2) in Fig. 1 of the main text. Figure 1d gives H_z phase profile at K corresponding to these two specific dots. Both states display typical vortex profiles centered at the honeycomb lattice. The counterparts at K' possess opposite chirality, as required by time-reversal symmetry. We will focus on the physics at K in the detail discussion, while that at K' can be derived from time-reversal symmetry.

The TE-like gap exists between the K point and the M point, which is highlighted as yellow region in Supplementary Figure 1b. Such indirect band gap will not affect on the K_1 valley bulk state and the valley-induced interface transport. Due to bulk-edge correspondence, the physics is completely same as previously well-known valley states as

the nontrivial topology of the gap is guaranteed by the bands below, i.e. TE1 band. However, the indirect gap will cause some minor issue (e.g. multi-mode effect) when the K_2 bulk state is excited, which is not considered in this work.

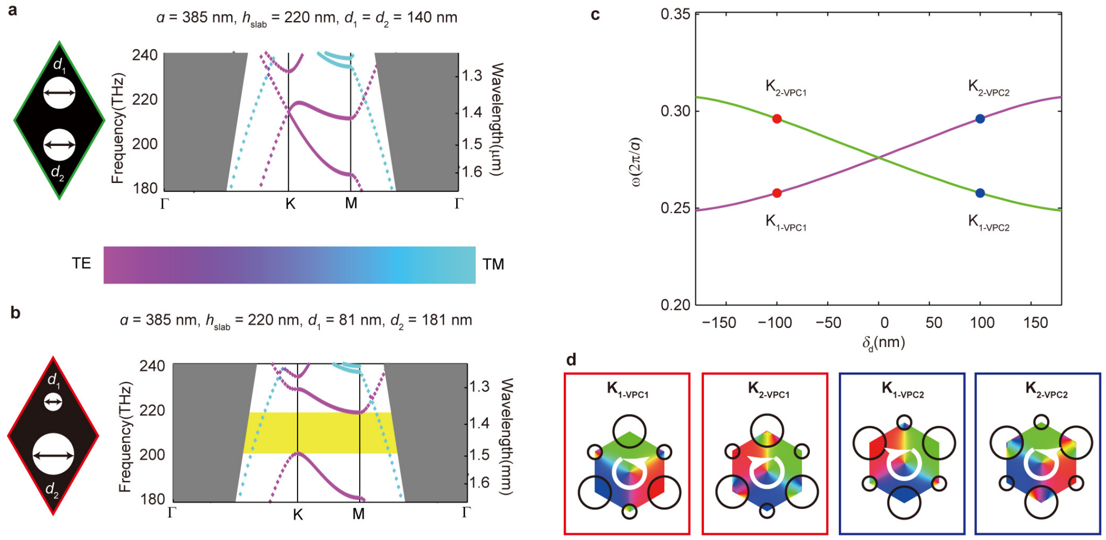

Supplementary Figure 1 Transition of valley topological phase with spatial-inversion-symmetry broken. **a-b**, Bulk band structures for δ_d = 0 nm ($d_1 = d_2$ = 140 nm) and δ_d = -100 nm (d_1 = 81 nm and d_2 = 181 nm), respectively. Here, $\delta_d = d_1 - d_2$ is the diameter difference between the upper and lower holes. The colormap indicates the linear polarization of photonic band. Yellow region: TE-like bandgap in calculation. Gray region: light cone of silica. **c,** Phase diagram revealed by the band-edge frequencies at K_1 and K_2 points. Purple/green curve implies the phase vortex locking with anticlockwise/clockwise. Red (blue) dots: the designed VPC1 (VPC2) in the main text. **d**, H_z phase profile at K_1 and K_2 points corresponding to the specific dots in **c**. Both states display typical vortex profiles centered at the honeycomb lattice.

Supplementary Note 2: Photonic valley Hall effect in silicon-on-insulator valley photonic crystal

In this section, we will show how to distinguish TE1 bulk states between K valley and K’ valley, originating from valley-chirality-locked property [see Figs. 1d-1e of the

main text]. Consider a regular triangle structure (with side length l = 30a) arranged by VPC1. Supplementary Figure 2a gives the schematic view of selective excitation of bulk states. The K/K' valley state separately propagates along the red/blue path, which is excited by right/left-circular polarized (RCP/LCP) source. Such phenomenon is called photonic valley Hall effect (PVHE), as an analog of the valley Hall effect where valley-polarized electrons move in opposite directions [2,3]. To confirm it, we illustrate the total-energy intensity of the excited K' (left) and K (right) valley in Supplementary Figure 2b. The LCP and RCP chiral sources are applied at λ = 1500 nm, respectively. The normalized operation frequency is slightly below that of $K_{1\text{-VPC1}}$ to guarantee the group velocity of propagating wave in bulk VPC as a non-zero value. Note that the region outside triangle structure is filled with silicon-rich nitride (n = 2.46) in order to match the parallel wavevectors at the interface between VPC and outside SiN waveguide [4].

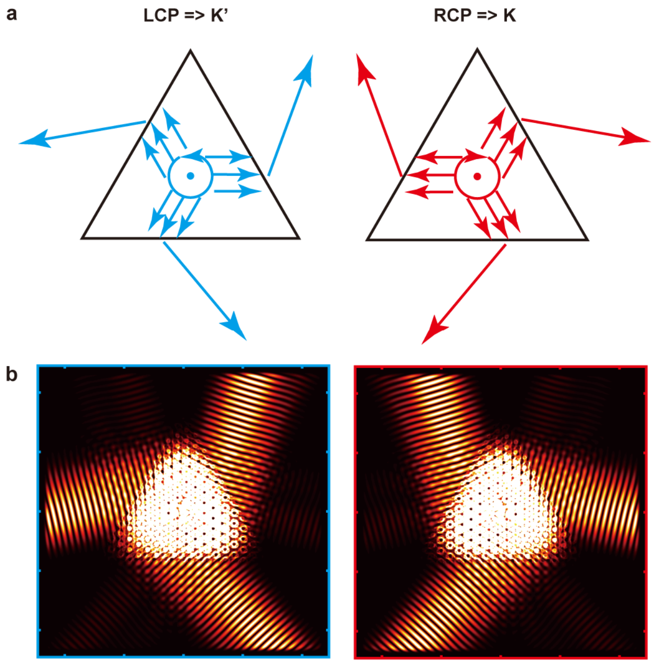

Supplementary Figure 2 Photonic valley Hall effect of silicon-on-insulator valley photonic crystal excited by circular-polarized dipole source. **a**, Schematic view of photonic valley Hall effect (PVHE). The K (K') valley state is selectively excited by a dipole source, where is placed at the center of regular triangle structure. **b**, Simulated total-energy intensity of the excited K' (left) and K (right) valley when applying the LCP and RCP chiral sources at $\lambda = 1500$ nm.

Supplementary Note 3: Valley-dependent topological edge states

As shown in Fig. 2c, the dispersion of the bearded-stack interface includes one with negative velocity at the K valley and the other with positive velocity at the K' valley. To insightfully observe the valley-dependent property, we also give the Poynting vectors and H_z phase of the edge states at K and K' valley in the left and right panels of Supplementary Figure 3, respectively. The left-forward chiral edge state is locked to clockwised phase vortex at K valley, while the right-forward K' valley mode is locked to anti-clockwised phase vortex. Such chiral-flow modes can be simplified as the illustration of Fig. 3a, implying the capability to unidirectionally excite by using single circular-polarized source.

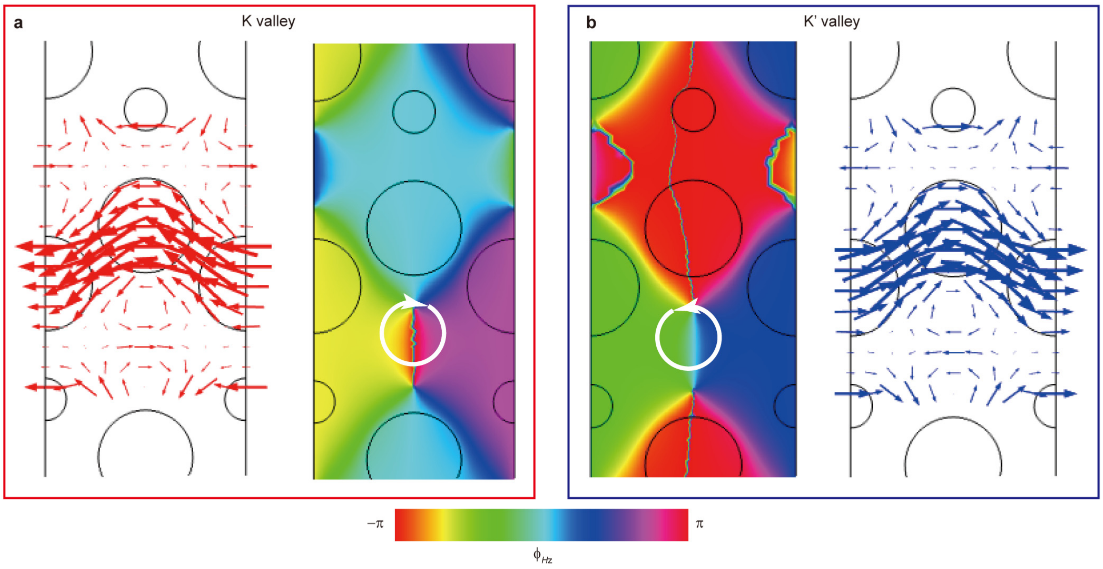

Supplementary Figure 3 Poynting vectors and H_z phase of valley-dependent topological edge states at **a**, K and **b**, K'. The left-forward chiral edge state is locked to clockwised phase vortex at K valley, while the right-forward K' valley mode is locked to anti-clockwised phase vortex.

The most ideal case for demonstration of valley-dependent robustness is the interface with 120° detour. From physics point of view, the 120° detour can guarantee the nontrivial topology originated from C_{3v} crystalline symmetry, so that it can avoid pseudospin (here referred to valley) flip to its time reversal partner. Therefore, inter-valley scattering is suppressed and the robustness occurs in valley-protected photonic crystal waveguide. From application point of view, we also studied other imperfect cases such as non-120° detour angles or real scatters. The pseudospin pair will flip to each other and lead to little scattering, but one may still observe broadband high transmission as the valley protection is "robust" to disorder-induced scattering. For example, the high-transmission plateau survives in 60° detour waveguide (Supplementary Figure 4a). For real scatters, the transmission has a little effect compared to the non-defect case when

introducing three scatters by filling the air holes with silicon (Supplementary Figure 4b). A more practical case is illustrated in Supplementary Figure 4c. We introduce random bias of up to 10% in each air-hole diameter, and perform 60 different sets of simulations using 3D FDTD calculator. One can still find that broadband high transmission plateau is again, robust to those random bias settings (purple curves in Supplementary Figure 4c).

In principle, there exists a little TE/TM coupling in our asymmetric slab. But the TE/TM coupling is too weak to affect valley-dependent interface transport. A direct evidence is that one can still observe the high-transmission plateau in the cases of some scatters (Supplementary Figure 4b) and random-bias diameters (Supplementary Figure 4c), as such high-transmission plateau is dominant for TE-like edge states. If the TE/TM coupling is strong enough, the valley-dependent edge states will leak to bulk photonic crystals and seriously reduce the high-transmission plateau. In general, one may use the eigenvalue of $z = 0$ mirror-flip operation P_z to characterize the strength of TE/TM coupling, where $P_z = +1$ and -1 for true z-even and z-odd eigenstates[4]. As for valley-induced edge transport near K/K' point, P_z is up to 0.97 (i.e. TE/TM energy ratio is $\left(1+P_z\right)^2/\left(1-P_z\right)^2 = 4312:1$). Such protection of close-to-one z parity makes TE-like guided mode difficult couple to TM polarization during interface propagation, even with scatters.

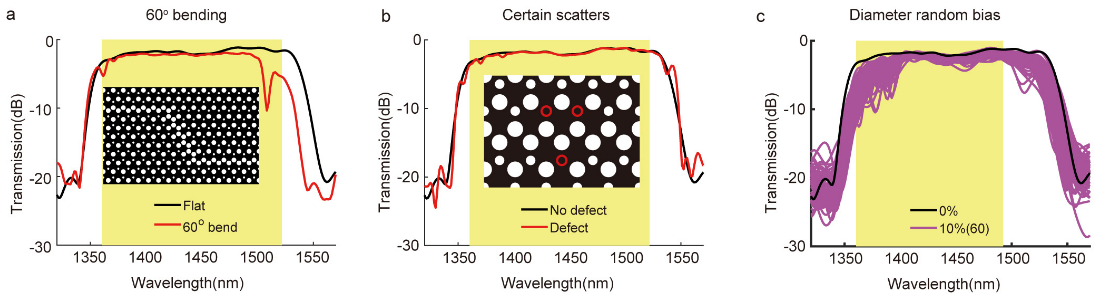

Supplementary Figure 4 Robustness of valley-dependent topological edge states. Full wave three-dimensional simulations on transmission spectra for **a**, 60° bending geometry, **b**, three scatters configuration through filling the air holes with silicon and **c**, 60 sets of 10%-random-bias configurations. Yellow region implies the TE-like bandgap in calculation.

Supplementary Note 4: Optical characterization setup

The experimental setup of optical characterization is shown in Supplementary Figure 5. The tunable lasers (Santec TSL-550/710) were used to generate continuous waves at telecom wavelength (1260 nm ~ 1640 nm). The incident light was firstly launched into a fiber polarization controller to select TE wave, and then coupled to the input waveguide with the aid of a polarization maintaining lensed fiber. A 1.7-μm-width silicon waveguide along the x direction was placed at the left end of the topological interface to launch TE-polarized waves into VPC device. After passing through the VPC device, the propagating wave coupled to the output waveguide at the right end of the interface and then collected by another lensed fiber. Then the signals were detected by an optical power meter (Ophir Nova-II) and the corresponding transmission spectra were retrieved with tuning the operation wavelength of the excited waves. On the other hand, some of scattered light turned to be out-of-plane radiation and was collected by a 20X microscope objective and then imaged by using an InGaAs CCD (Xenics Bobcat-640-

GigE). Such vertical path was used to monitor the couple between lensed fiber and input/output waveguide. All the transmission spectra are normalized to the 1.7-μm-width silicon strip waveguide located in the same writing field near the VPC devices.

As for the photonic routing experiment, the experimental setup was quite the same, only different in the output part. The incident light is also generated by the tunable lasers and then coupled to the sample with the aid of a polarization maintaining lensed fiber, after passing through the fiber polarization controller to select TE mode. There were two silicon waveguides at the left of the sample. When the incident light couple to the left/right input waveguide, it will partly convert to LCP/RCP at the designed-mircodisk, then the remaining LCP/RCP light will pass through the left/right channel. Another silicon waveguides were fabricated at the end of each channel, and the propagating wave coupled out in the *z*-direction thanks to the gratings at the end of the waveguide. The out-of-plane radiation was collected by a 20X microscope objective and then imaged by using an InGaAs CCD. After the appropriate adjustment of the exposure time, the intensity of each end of the channels was gained from the CCD and the ratio rate can be calculated. The full-band ratio rate can be measured by tuning the operation wavelength of the excited waves.

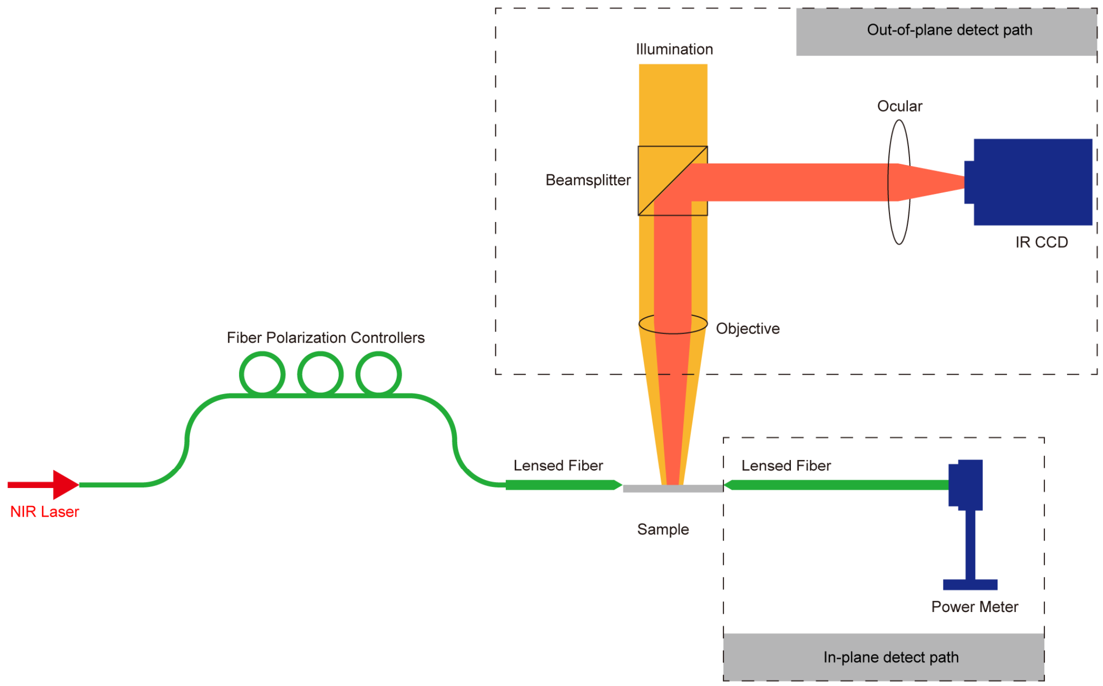

Supplementary Figure 5 Schematic diagram of experimental setup for transmission measurements and optical microscope images. A NIR continuous wave was firstly launched into a fiber polarizer to select TE mode, and then coupled to the device with the aid of lensed fiber. After passing through device, the output signals can be divided into two parts: one is the in-plane propagating wave collected by another lensed fiber and detected by an optical power meter, the other is the out-of-plane radiation collected by a 20X microscope objective and then imaged by using an InGaAs CCD. Another purpose of out-of-plane detect path is to monitor the couple between the lensed fiber and the input/output waveguide. For the routing images measurement (Fig. 4), the in-plane detect path was removed, as the in-plane propagating waves can be turn into out-of-plane radiation by using grating couplers.

Supplementary Note 5: Details for photonic routing with a subwavelength microdisk

In this section, we will show more details about the experiments of photonic routing devices. The far-field microscope images in the second row of Supplementary Figure 6 have demonstrated in the main text, implying that the high-directional topological routing effect exists in silicon-on-insulator valley photonic crystal. Here, we offer more routing images at out-of-bangap wavelengths both for topological and normal routing devices, respectively. For topological routing, the images did not preserve unidirectional coupling

outside the bandgap (see the first and third rows of Supplementary Figure 6), due to the simultaneous excitation of additional bulk states. For normal routing, the guided interface using strip waveguide is insensitive to the optical vortex of microdisk, in correspondence with low-directionality spectra. Note that the scattering spot near sample is mainly caused by out-of-plane scattering among input waveguide, subwavelength microdisk and SOI VPC. It is clear that the spots output from grating couplers have a lower intensity relative to that of the central excitation spot. In spite of this side effect, the valley-dependent directional emission is already visible to be distinguished. Supplementary Figure 7a and 7b show measured (also in Fig. 4h) and simulated directionality spectra for topological photonic routing devices. Simulated directionality spectra are in good agreement with measured results, except for the blue shift of the peak owing to fabrication error.

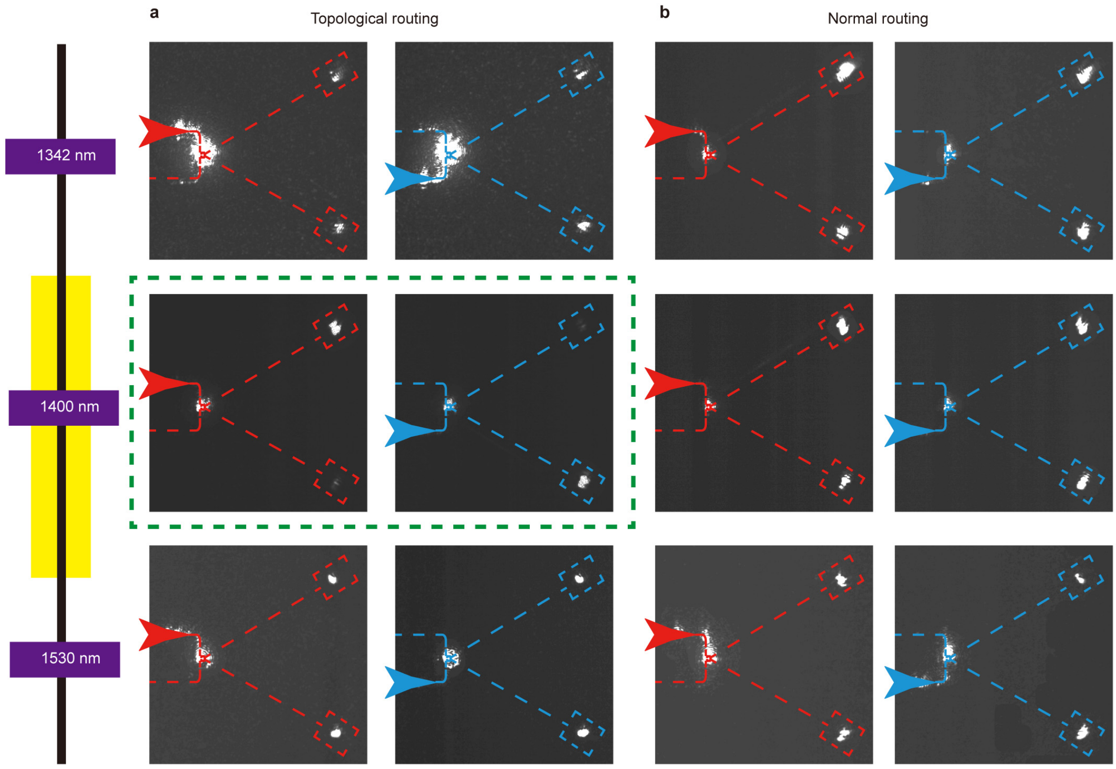

Supplementary Figure 6 Photonic routing profiles measured by an optical far-field microscopy (20X objective). **a**, topological routing device and **b**, normal routing device with tuning the operation wavelength at λ = 1342 nm, 1400 nm and 1530 nm, respectively **.** Yellow region implies the TE-like bandgap in calculation. Red dashes indicate WVG1 incident cases while blue dashes are for WVG2 incidence.

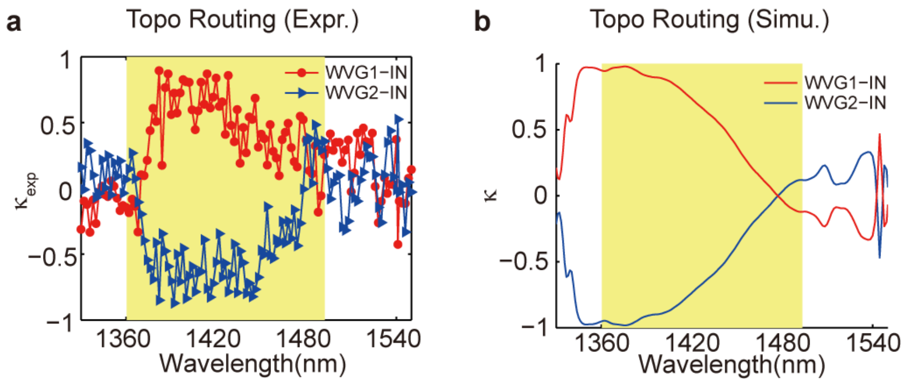

Supplementary Figure 7 Measured and simulated directionality spectra for routing devices. **a,** Measured directionality as a function of operation wavelength for topological routing device, which has been shown clearly in Fig. 4h. **b**, Simulated directionality spectra are in good agreement with measured results, except for the blueshift of the peak owing to fabrication error. Yellow region implies the TE-like bandgap in calculation.

Supplementary References

1. Xiao, D., Yao, W. & Niu, Q. Valley-contrasting physics in graphene: magnetic moment and topological transport. *Phys. Rev. Lett.* **99**, 236809 (2007).
2. Chen, X.-D., Zhao, F.-L., Chen, M. & Dong, J.-W. Valley-contrasting physics in all-dielectric photonic crystals: Orbital angular momentum and topological propagation. *Phys. Rev. B* **96**, 020202 (2017).
3. Dong, J.-W., Chen, X.-D., Zhu, H., Wang, Y. & Zhang, X. Valley photonic crystals for control of spin and topology. *Nat. Mater.* **16**, 298-302 (2017).
4. Joannopoulos, J.D., Johnson, S.G., Winn, J.N. & Meade, R.D. Photonic crystals: molding the flow of light. (Princeton university press, 2011).
